# Attention-enhanced corn disease diagnosis using few-shot learning and VGG16

**DOI:** 10.1016/j.mex.2025.103172

**Published:** 2025-01-15

**Authors:** Ruchi Rani, Jayakrushna Sahoo, Sivaiah Bellamkonda, Sumit Kumar

**Affiliations:** a Department of Computer Science and Engineering, Indian Institute of Information Technology Kottayam, 686635, Kerala, India; b Department of Computer Engineering and Technology, School of Computer Engineering and Technology, Dr.Vishwanath Karad MIT World Peace University, Pune, 411038, Maharashtra, India; c Symbiosis Institute of Technology, Pune Campus, Symbiosis International (Deemed University), Pune, Maharashtra, 412115, India

**Keywords:** Corn, Plant disease detection, Few shot Learning, Prototypical networks, Attention mechanism, VGG16, VGG16 integrated Attention Mechanism and Prototypical Few-Shot Learning for Corn Disease Classification

## Abstract

Plant Disease Detection in the early stage is paramount. Traditionally, it was done manually by the farmers, which is a laborious and time-intensive task. With the advent of AI, Machine Learning and Deep Learning methods are used to detect and categorize plant diseases. However, they rely on extensive datasets for accurate prediction, which is impracticable to acquire and annotate. Thus, Few Shot Learning is the state-of-the-art model in machine learning, which requires minimum examples to train the model for generalization. As humans need a few examples to recognize things, Few-shot Learning mimics the same human brain process. The proposed work uses a pre-trained convolution neural network, VGG16, as the backbone, fine-tuned on the corn disease dataset. An attention module is integrated with the backbone, and further, prototypical few-shot learning is used for corn disease prediction and classification with an accuracy of 98.25 %.

• The proposed model intends to identify the diseases early, so the insights generated would be relevant for farmers, and probable losses can be reduced.
• By applying Few-Shot Learning, the system avoids the significant challenges of requiring extensively annotated datasets, making it feasible for real-world agricultural applications.
• Incorporating a fine-tuned VGG16 backbone along with an attention mechanism and prototypical Few-Shot Learning results in a robust and scalable solution with high accuracy for classifying corn diseases.

## Graphical abstract

**Figure:**
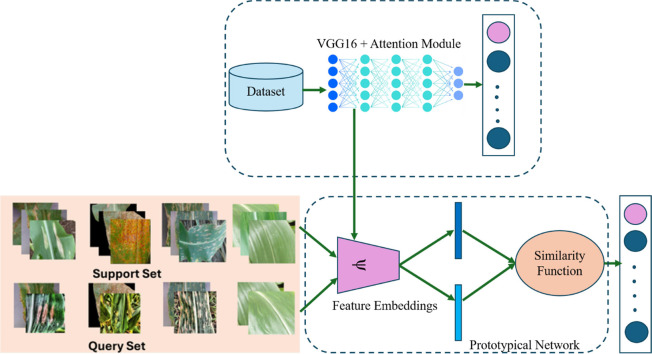

Specifications table

**Table:** 

Subject area:	Machine Learning, Deep Learning, Agricultural Informatics
More specific subject area:	Corn Disease Detection, VGG16, Attention Mechanism, Few-Shot Learning
Name of your method:	VGG16 integrated Attention Mechanism and Prototypical Few-Shot Learning for Corn Disease Classification
Name and reference of original method:	NA
Resource availability:	Google Colab, Python, PyTorch

## Background

Maize, or corn, is one of the most important cereal crops globally, playing a pivotal role in food security, animal feed production, and industrial applications. Corn is an essential food in many regions of the world, and it is consumed in the form of popcorn, cornflakes, tacos, bread, etc. In industry, corn produces biodegradable plastic, paper, textiles, and ethanol (biofuel) [1]. The largest producer of corn is the United States, followed by China, Brazil, Argentina, Ukraine, and India [2]. However, the cultivation of maize is constantly challenged by various diseases that can lead to substantial yield losses and compromise crop quality. Timely and accurately identifying these diseases is crucial for implementing effective disease management strategies and ensuring sustainable maize production [[3], [4], [5], [6], [7]].

Traditional disease detection methods in maize crops are often exhausting and impractical due to the time-consuming process involving visual inspection by agricultural specialists, especially for large farmlands. Additionally, the traditional methods may struggle with the dynamic nature of the disease outbreak due to variability in climate and much less scalability for larger farms. Therefore, there is an urgent need for fast, automated, and efficient disease classification systems that can rapidly and accurately classify maize diseases.

Deep learning techniques have recently taken the computer vision field to a new level, providing significant tools for image recognition and classification tasks. However, it is common that deep learning models require large amounts of labeled data for training, which can be challenging, especially in agricultural domains where labeled datasets are often limited and imbalanced. Detection and classification of diseases in maize plants are important tasks in precision agriculture, which can increase crop productivity and economic returns by reducing harmful chemicals. Recent technological advancements, specifically deep learning-based approaches, have contributed significantly to the automation of plant disease detection systems. Sharma et al. presented a new ClGan (Crop Leaf Gan) framework along with ClGanNet for maize leaf disease classification [8]. Their approach addresses the challenges of blurred background information and data imbalances, achieving high accuracy while using fewer parameters than existing methods. Enlin et al. proposed the MDCDenseNet model for maize disease identification, combining DenseNet121 with a multi-dilated module and convolutional block attention mechanism [9]. Their method outperformed other methods, especially on field-collected datasets with complex backgrounds. Various research studies proposed new deep-learning architectures for maize disease detection, including SKPSNet-50, cascaded CNN models, MResNet, MMF-Net, and end-to-end models using EfficientNetB0 and DenseNet121 [[10], [11], [12], [13], [14], [15]]. These approaches use recent advances in feature extraction and classification techniques to achieve high accuracy in disease classification. Phan et al. used SLIC segmentation for super-pixel creation and deep learning models such as VGG16, ResNet50, and DenseNet121 for maize disease identification [10]. Their approach demonstrated promising results in accurately identifying diseased regions on maize leaves. Ahmad et al. checked how the deep learning models generalize from one dataset and environment to another in maize disease identification [16]. The results show that with the help of transfer learning and data augmentation, model generalization can be significantly improved, and their performance enhanced for better field condition outcomes. The literature review focuses on the diversity of deep learning approaches applied to maize disease detection, with unique contributions towards improved accuracy, efficiency, and generalization capabilities. These developments indicate the increasing importance of deep learning in solving the challenges of precision agriculture and crop disease management. Naik et al. aim to enhance the performance of FSL in classifying primitive paddy varieties by combining 2D discrete wavelet transform (2D-DWT) features with CNN deep features [17]. They optimized the hyperparameters of CNN using the Grey Wolf Optimizer, which significantly improved the recognition rate on limited training data. This work shows the potential of integrating advanced feature extraction methods and optimization algorithms to improve the effectiveness of FSL in agricultural contexts.

Detection of corn diseases through developing deep learning models has recently become an important subject in agriculture and food security issues [[18], [19], [20], [21], [22], [23], [24]]. Approaches for enhancing real-world accuracy, efficiency, and adaptability have been proposed. Zeng et al. presented a lightweight, dense-scale network, LDSNet, with 95.4 % accuracy on real-world corn leaf disease images [24]. This model uses dense dilated convolutions to enhance scale adaptability while reducing the number of parameters, making it more efficient. However, LDSNet has limited comparative analysis with recent state-of-the-art models, which restricts its broader applicability in diverse datasets and scenarios. Another notable approach is by Divyanth et al., who proposed a two-stage semantic segmentation pipeline using UNet and DeepLabV3+ architectures [25]. Their approach attained a mean weighted Intersection over Union (mwIoU) of 0.9422 for UNet and 0.7379 for DeepLabV3+ on a custom corn disease dataset. Though this approach stresses the detailed estimation of disease severity, its computational complexity makes it difficult to be deployed in real-time in resource-constrained environments. Other researchers have emphasized the development of lightweight models to improve adaptability across multiple crop types. For example, Verma et al. presented a lightweight CNN model that obtained 99.74 % accuracy for corn diseases [26]. However, the model decreased performance in detecting diseases in other crops, such as rice, and, therefore, has a low generalization capability. Advanced architectures like TreeNet, which combines the feature fusion of Darknet-53 and DenseNet-201, obtained an impressive accuracy of 99.8 % on the Plant Village dataset [27]. Although the performance of TreeNet is high, its computational cost remains a significant limitation, especially for real-time or edge-computing applications. Hybrid and transformer-based approaches have also been promising in enhancing disease detection. For example, 3DCNN-RNN hybrid models, Whale Optimization Algorithms, and transformer-based architectures such as MaxViT have improved accuracy and robustness. These methods suffer from computational complexity, limited interpretability, and lack of generalization to common datasets [28,29]. Waheed et al. recently proposed an optimized DenseNet architecture for corn leaf disease recognition with an impressive accuracy of 98.06 % [30]. This model outperforms traditional CNNs such as VGG19 and EfficientNet in accuracy while using fewer parameters and fewer computations. However, Yang et al. recently developed the EIRP model by applying machine learning to predict southern corn rust risks in northern China from meteorological data and infection dynamics [31]. The precision rate of the model is 93.51 %, which highlights its effectiveness in airborne disease prediction, as validated by observational data from 66 maize-growing regions. In addition, Antolinez et al. studied the capability of NIR images acquired by UAVs in conjunction with transfer learning-based CNNs for maize fungal disease classification [32]. This new approach showed an accuracy level of 86.7 % and proved the NIR images in accurate disease classification ability along with GPS data in the targeted application of fungicides. Therefore, these studies focus on applying advanced deep learning and predictive modeling techniques to tackle the diseases in corn and propose solutions that achieve the highest possible accuracy with a reasonable degree of ecological sustainability.

This study attempts to bridge the gap between conventional disease classification approaches by exploiting state-of-the-art machine learning algorithms in precision agriculture. This paper proposes a new approach to classifying corn diseases using few-shot learning combined with a meta-learning framework. Few-shot learning enables the model to generalize well to new, unseen disease instances with minimal labeled examples, thereby reducing dependence on highly annotated datasets. The proposed model can learn from diverse maize disease instances and adapt rapidly to new disease types or environmental variations during deployment by incorporating meta-learning techniques. The significance of this work is that it allows for a scalable and efficient approach to maize disease classification, which is especially helpful in areas with limited access to large, annotated datasets. Also, the flexibility of the proposed framework is likely to allow its extension into other crops or agricultural areas with similar problems. This adaptability is important to address upcoming disease threats and possible impacts of changing environmental conditions on crop health. Furthermore, adding attention mechanisms to the framework enables the model to better focus on the most relevant regions of the input images, like diseased areas on corn leaves. The attention module refines feature extraction by generating and scaling attention maps emphasizing localized and global features necessary for accurate diagnosis. This refines the model to detect subtle patterns that might not be detected using traditional methods, improving generalization in few-shot learning scenarios. This work enlightens the advantages of few-shot learning in agricultural applications and introduces a novel framework with adaptability, efficiency, and scalability. Additionally, by addressing these challenges, the proposed work will be helpful in the advancement of precision agriculture, enhancement of the resilience of maize production systems against disease outbreaks, and setting the stage for future innovations in automated crop disease management.

Table 1 compares the different models and methodologies applied to detect corn disease, focusing on datasets, performance metrics, specific features, and main drawbacks that describe the achievements and limitations in this area. This comparison highlights the necessity of balanced approaches that achieve high accuracy while addressing practical concerns such as computational efficiency, scalability, and generalization across diverse datasets.

**Table 1:** Comparison of different corn disease detection models.

Model/Methodology	Dataset Used	Results	Key Features	Limitations	Ref
LDSNet (Lightweight dense-scale network)	Real-world corn leaf disease images	Accuracy: 95.4 %	Dense dilated convolution for scale adaptability, reduced parameters	Limited comparison with newer models	[24]
Two-stage semantic (UNet, DeepLabV3+)	Custom dataset	mwIoU (UNet): 0.9422, mwIoU (DeepLabV3+): 0.7379	Focus on disease severity estimation	Computational complexity in real-time scenarios	[25]
Lightweight CNN for corn, rice, and wheat diseases	Multiple crops (Corn, Rice, Wheat)	Accuracy (Corn): 99.74 %, Overall: 84.4 %	Lightweight, single model for multiple crops	Lower performance for rice disease	[26]
TreeNet with feature fusion (Darknet-53, DenseNet-201)	Plant Village dataset	Accuracy: 99.8 %	Robust preprocessing, integrated models	High computational cost	[27]
Hybrid 3DCNN-RNN with Whale Optimization	Maize_in_field, KaraAgro AI maize	Accuracy: >90 %	Temporal and spatial feature extraction, hyperparameter optimization	Limited datasets, lacks comparison with newer methods	[28]
MaxViT vision transformer with SE block	PlantVillage, PlantDoc, CD&S dataset	Accuracy: 99.24 %	Vision transformer with advanced normalization, large dataset	High model complexity	[29]
Optimized DenseNet	Corn leaf disease dataset	Accuracy: 98.06 %	Lightweight model, optimized for speed	Comparison with older CNN models	[30]
Ecoclimatic index risk prediction (EIRP) model	Shandong Province (15-year meteorological data)	R² (Testing): 0.84	SCR prediction based on spore migration and weather data	Specific to SCR, limited geographic application	[31]
Transfer learning CNN	UAV infrared images	Accuracy: 86.7 %	NIR images for fungal detection	Lower accuracy than newer methods	[32]

## Method details

**Fig. 1:**
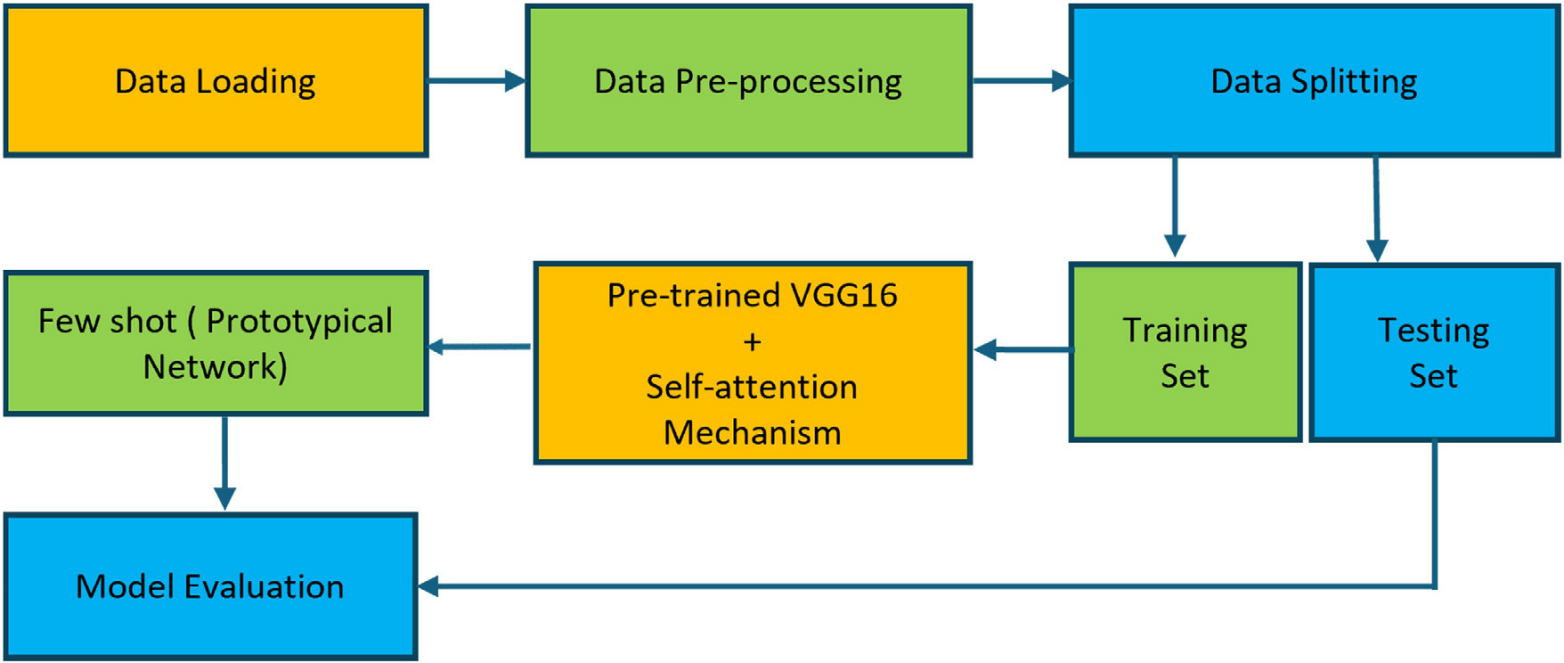
Methodology of the proposed work.

The proposed methodology for corn disease diagnosis integrates an attention-enhanced approach with few-shot learning and the VGG16 architecture, as shown in Fig. 1. The process begins with data loading, where the dataset of corn diseases is imported for analysis. After that, data pre-processing is performed to standardize the input images, which makes them ready for training. The data is divided into a training set and a testing set. This training set is used to learn the model, and the testing set is held aside for the performance evaluation process. A pre-trained VGG16 model with enhanced self-attention mechanisms extracts meaningful features from the input images. During model training, self-attention mechanisms enhance the model's ability to focus on important regions in the images that can provide more accurate feature learning. The embeddings of the images in support set/training data are extracted, which goes to pre-trained VGG16 and then to the attention modules, and the prototype is calculated using the prototypical few shots learning technique. Finally, the distance between the prototype and the query set/testing set image is calculated, and based on this distance, images are classified into different classes. The framework is validated to ensure the classification of corn disease categories under few-shot learning scenarios.

### Corn diseases

**Fig. 2:**
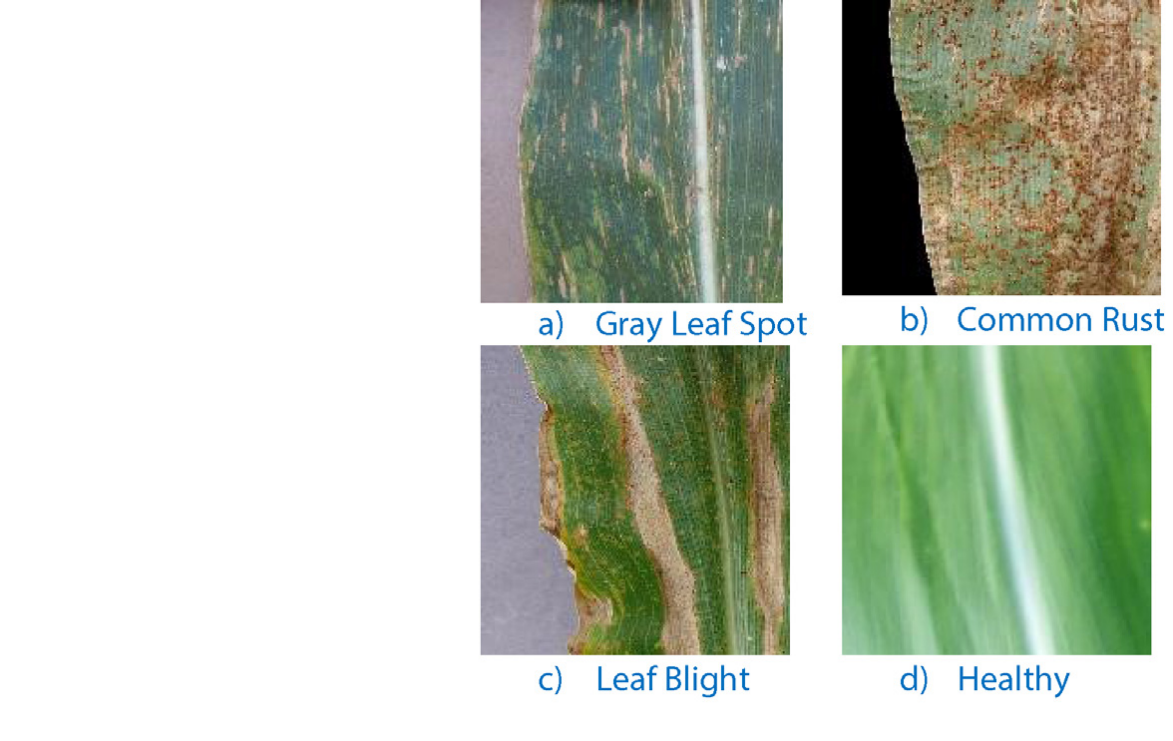
Corn diseases.

Corn (maize) is a vital crop globally, susceptible to various diseases that significantly impact yield and quality. The most common corn diseases include Corn Blight, Corn Common Rust, and Gray Leaf Spot, each caused by distinct pathogens and presenting unique symptoms. Fig. 2 illustrates examples of corn diseases, including (a) Gray Leaf Spot, (b) Common Rust, (c) Leaf Blight, and (d) Healthy leaves.

• Corn Blight: A fungal disease caused by Bipolaris maydis, it manifests as brown or gray lesions on the leaves, often reducing photosynthetic capability and weakening plant health.
• Corn Common Rust: Caused by Puccinia sorghi, it leads to reddish-brown pustules on leaves, disrupting the plant's ability to sustain growth.
• Gray Leaf Spot: Resulting from Cercospora zeae-maydis infection, it creates rectangular gray or brown lesions that can merge, leading to severe leaf tissue loss.

### Data collection and preprocessing

The corn/maize dataset used in this study is taken from Roboflow Universe and Kaggle [33,34]. The dataset contains images of three corn diseases: leaf blight, common rust, and gray leaf spot, along with healthy corn images. By combining these four classes, a dataset of 1320 images is prepared. Model generalization can be improved by merging datasets from different sources because data representation may vary in every dataset. This diversity includes variations in lighting, angles, and background conditions, making the trained model more robust to these differences. Various basic data augmentation techniques, such as image preprocessing methods, are applied to improve the trained images' picture quality, further enhancing the model generalization. The training transformations included resizing the images to 224 × 224 pixels, random horizontal and vertical flips with 50 % probability, random rotations between -10 to +10 degrees, and color jittering. All images are then converted to tensors and normalized using mean and standard deviation values typical for pre-trained models.

### Backbone network

This research proposes an attention-enhanced method that integrates a self-attention mechanism with a VGG16 backbone, improving the diagnosis of corn diseases using few-shot learning. The pre-trained VGG16 model is the backbone network, leveraging its capacity to extract rich, high-dimensional features from input images. Originally trained on the ImageNet dataset, VGG16 is widely recognized for simple yet powerful architecture, built with 16 layers consisting of convolutional, pooling, and fully connected layers.

Convolutional layers composed of the backbone VGG16 have utilized small 3x3 filters with the activation ReLU, combined with the max-pooling layer that reduces the spatial dimension, preserving the critical information. These feature maps now proceed for full connection layers utilizing dropout, which prevents over-fitting to classes on the output layer and is further modified to comply with classes involved with the corn disease; this is fine-tuned on a relatively small dataset involving corn disease.

### VGG16 with attention mechanism

Feature learning is further enhanced in VGG16 architecture by incorporating attention mechanisms. This feature helps the model pay attention to the most significant parts of the input image that, in turn, aids it in detecting very fine patterns of diseased corn leaves. The integration follows the following key steps:

• Base VGG16 Model: The original convolutional layers of the VGG16 architecture remain intact for feature extraction.
• Attention Blocks: These blocks are strategically added after specific convolutional layers to refine the feature maps. Each attention block operates as follows:
  ○ Local Features: Initial convolutional layers capture localized features such as edges and textures.
  ○ Global Features: A global average pooling operation aggregates these local features into global representations.
  ○ Attention Feature Map: Attention maps are generated to diagnose the image's most prominent features or regions.
  ○ Refinement: The attention maps are scaled and combined with the original feature maps to produce refined outputs using element-wise addition.

Including the self-attention mechanism, the VGG16 model effectively identifies and emphasizes the diseased regions in the input images, leading to more accurate feature extraction. This enhanced focus is particularly valuable in few-shot learning scenarios where the dataset is small, as it improves the model's ability to generalize and diagnose corn diseases reliably.

### Few-shot learning

The few-shot learning strategy is drafted as an N-way K-shot classification task. This work employs a 4-way 5-shot strategy (Four classes with five examples per class). The training strategy involves episodic training, where each episode mimics a few-shot learning task. For each episode, a Support Set will be created, with K examples from each of the N classes and a Query Set with separate examples from the same N classes to evaluate the model. The prototype of each class of the support set is created from the input data. Then, the Euclidean distance of the embedding query set example and support set prototype is calculated. Based on the similarity, the query example is classified.

### Prototypical network algorithm

For computing the prototype $c_{m}$ for each class m in the support set S, the feature vectors of all examples x belonging to the class are averaged:

(1) $$c_{m}=\frac{1}{n}\sum_{x\in x_{p}}f\left(x\right)$$

After prototype computation, for each example$\;x_{q\;}$in the query set Q, the Euclidean distance is calculated from the class prototype $c_{m}$:

(2) $$d\left(x_{q},c_{m}\right)=|f\left(x_{q}\right)-c_{m}{|}_{2}$$

The image from the query set is classified based on the calculated distance to the closest prototype:

(3) $$\hat{y_{q}}=\text{argmi}n_{m}\;d\left(x_{q},c_{m}\right)$$

The softmax function converts the distance scores into probabilities, providing a categorical distribution over the possible classes for each query example:

(4) $$p\left(y=m|x_{q}\right)=\frac{\exp\left(-d\left(f\left(x_{q}\right),c_{m}\right)\right)}{\sum_{m^{\prime }}\;\exp\left(-d\left(f\left(x_{q}\right),c_{m^{\prime }}\right)\right)}$$

The training process employs a cross-entropy loss function for each few-shot task:

(5) $$L_{CE}=-\sum_{m=1}^{K}y_{m}\log\left(p\left(y=m|x_{q}\right)\right)$$

**Fig. 3:**
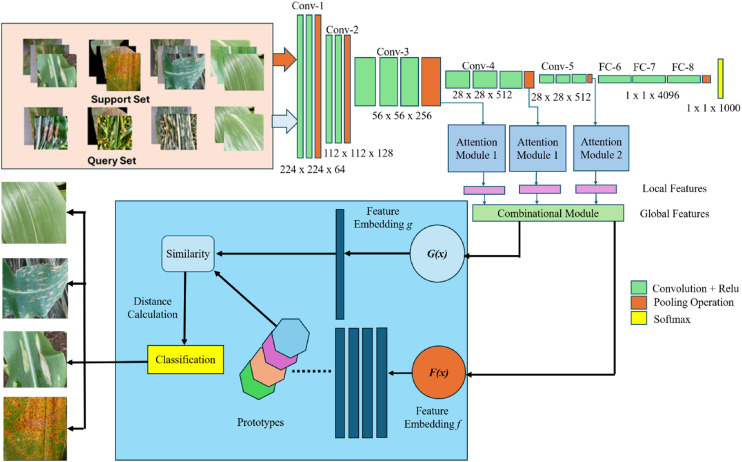
Proposed model architecture.

Fig. 3 shows the architecture of the proposed model having pre trained VGG16 as a backbone network integrated with attention modules, providing enhanced feature embeddings which are further used in a few-shot learning techniques to calculate the prototype of each class and based on the distance between the prototype and query set image embedding, the query set image is classified. The softmax function is used to find the probabilities of each class, and the cross-entropy loss function is used to calculate the loss, which shows the gap between predicted and actual probability distribution.

## Method validation

The proposed attention-enhanced VGG16 method for corn disease diagnosis with few-shot learning is implemented using the PyTorch deep learning framework. The model is optimized with the Adam optimizer using a learning rate of 0.001. A batch size of 32 is chosen to ensure training stability, and the cross-entropy loss is computed based on the distance to the class prototypes. Experiments are conducted on a workstation equipped with an NVIDIA RTX 3080 GPU.

**Fig. 4:**
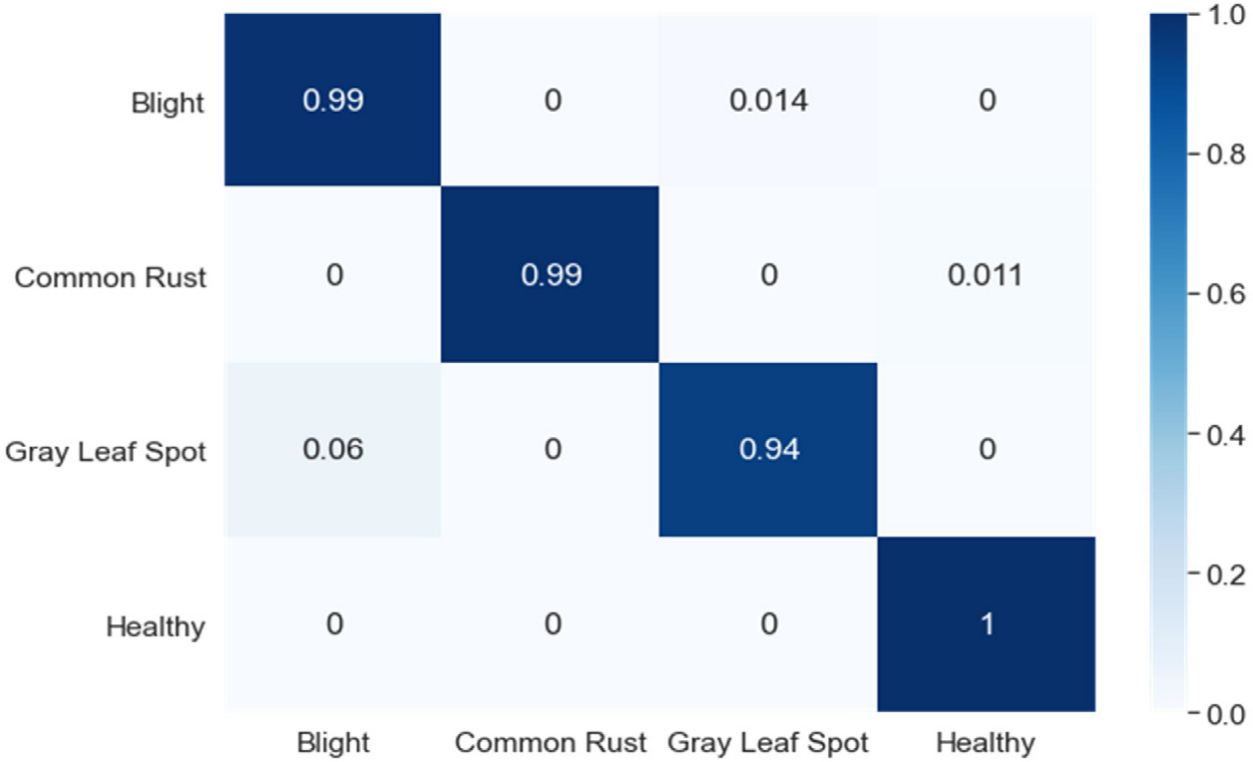
Confusion matrix of the model.

The model achieved a test accuracy of 98.25 % for 4-way 5-shot and demonstrated its effectiveness in few-shot learning scenarios. Various pretrained convolution neural networks are evaluated considering crucial performance parameters, such as accuracy, feature extraction capabilities, and the advantages of transfer learning. Consequently, VGG16 is chosen as a backbone network due to its high efficacy in extracting informative features from the data. The VGG16 model is trained for 30 epochs. During training, training loss and validation loss are measured. Fig. 4 presents the Confusion Matrix, showcasing the model's classification performance across disease categories. The matrix reveals that the model accurately identifies most disease categories, with minimal misclassifications observed in visually similar classes. For example, the model occasionally confuses Gray Leaf Spot and Common Rust, reflecting their overlapping visual features.

**Fig. 5:**
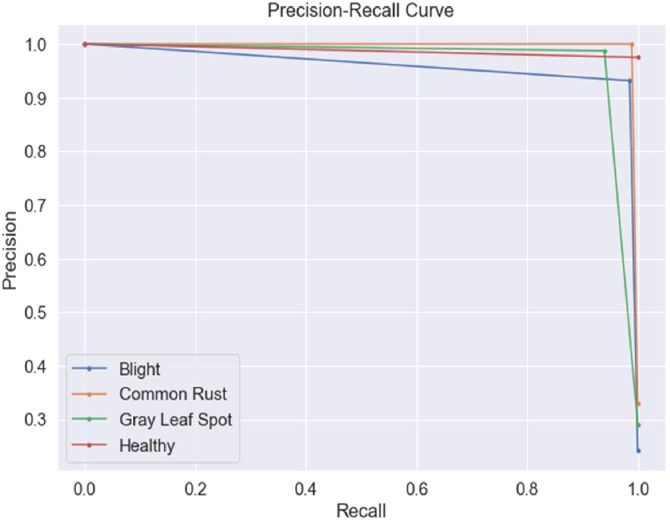
Precision-recall curve of the model.

Fig. 5 shows the Precision-Recall Curve (PRC), indicating the trade-off between precision and recall under class imbalance scenarios. The PRC demonstrates that the model maintains consistently high precision across varying recall levels, even for minority classes like ‘Healthy,’ which typically have fewer instances. This validates the model's robustness in handling imbalanced datasets.

**Fig. 6:**
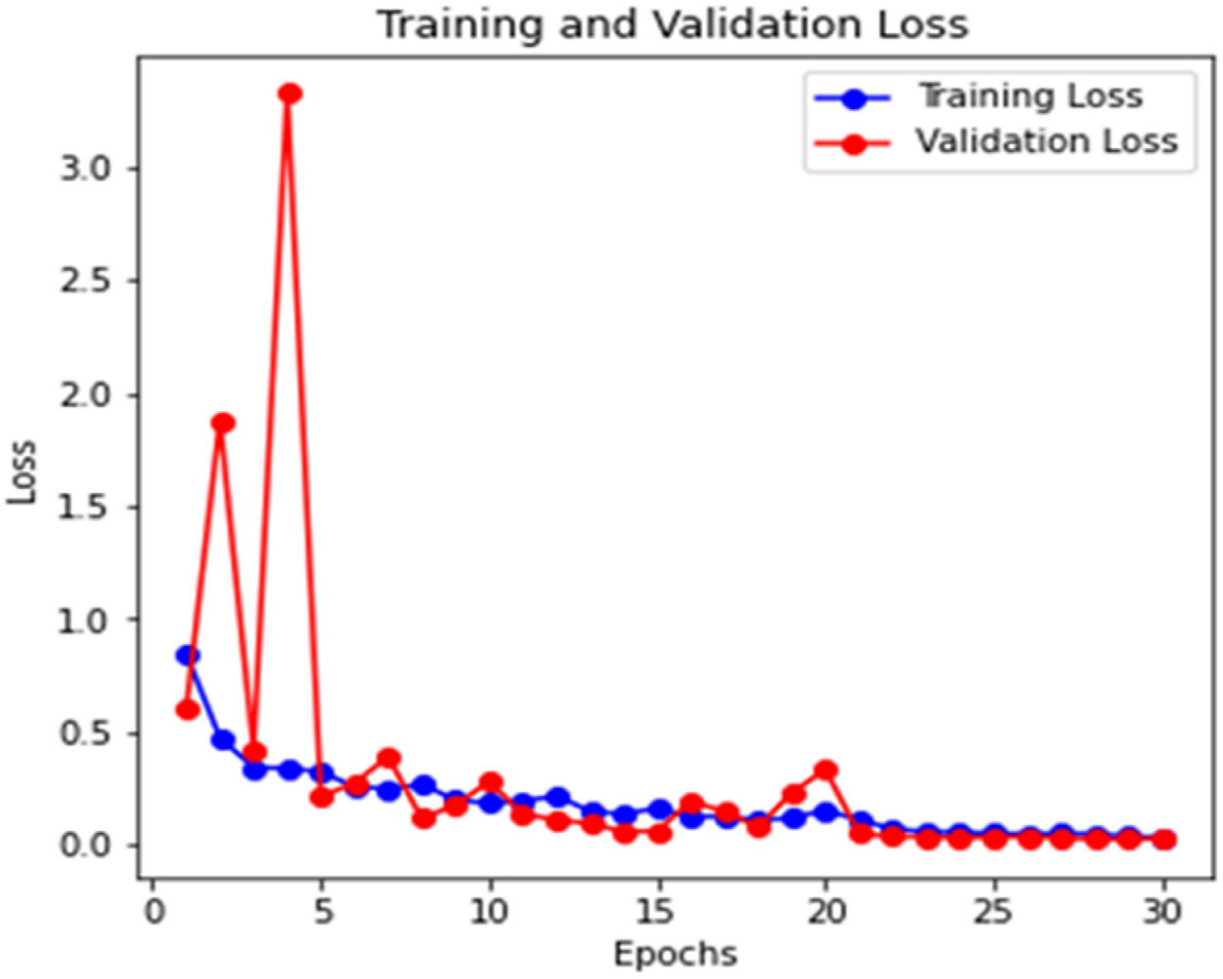
Training and validation loss of the model.

**Fig. 7:**
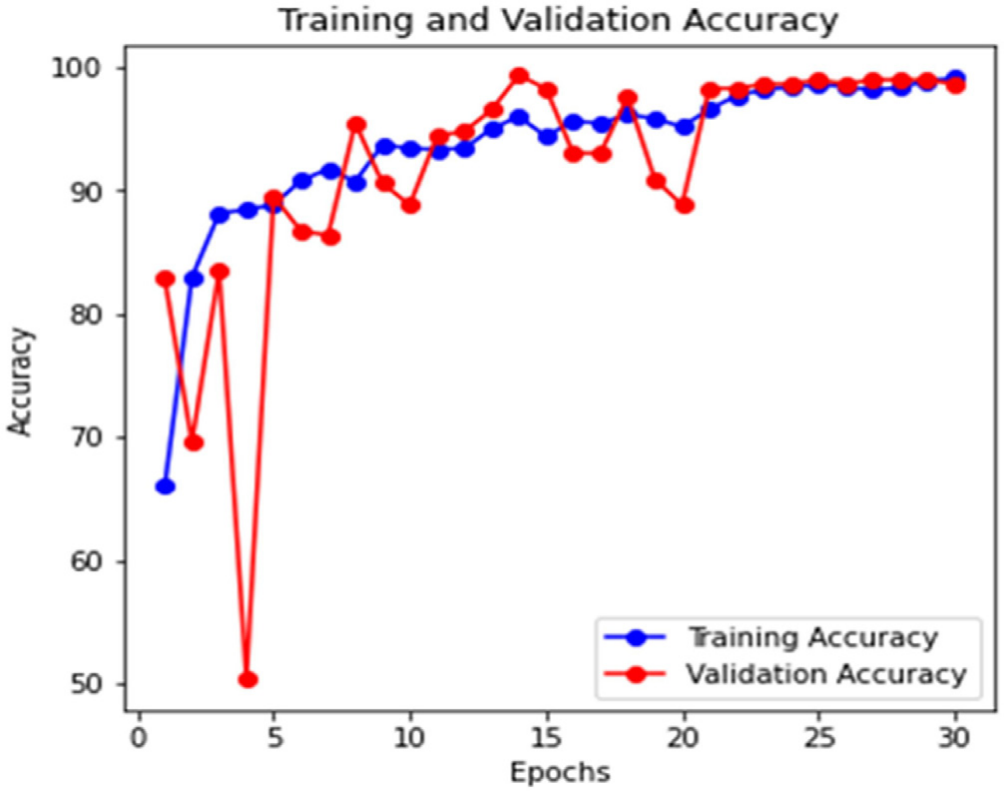
Training and validation accuracy of the model.

**Fig. 8:**
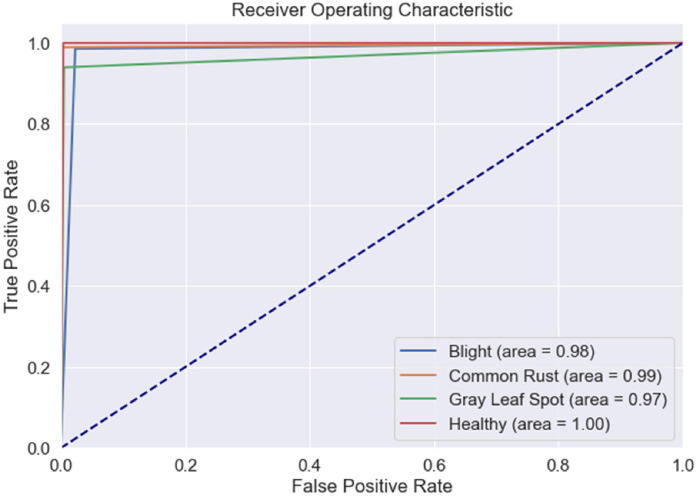
Receiver Operating Characteristic (ROC) curve of the model.

**Fig. 9:**
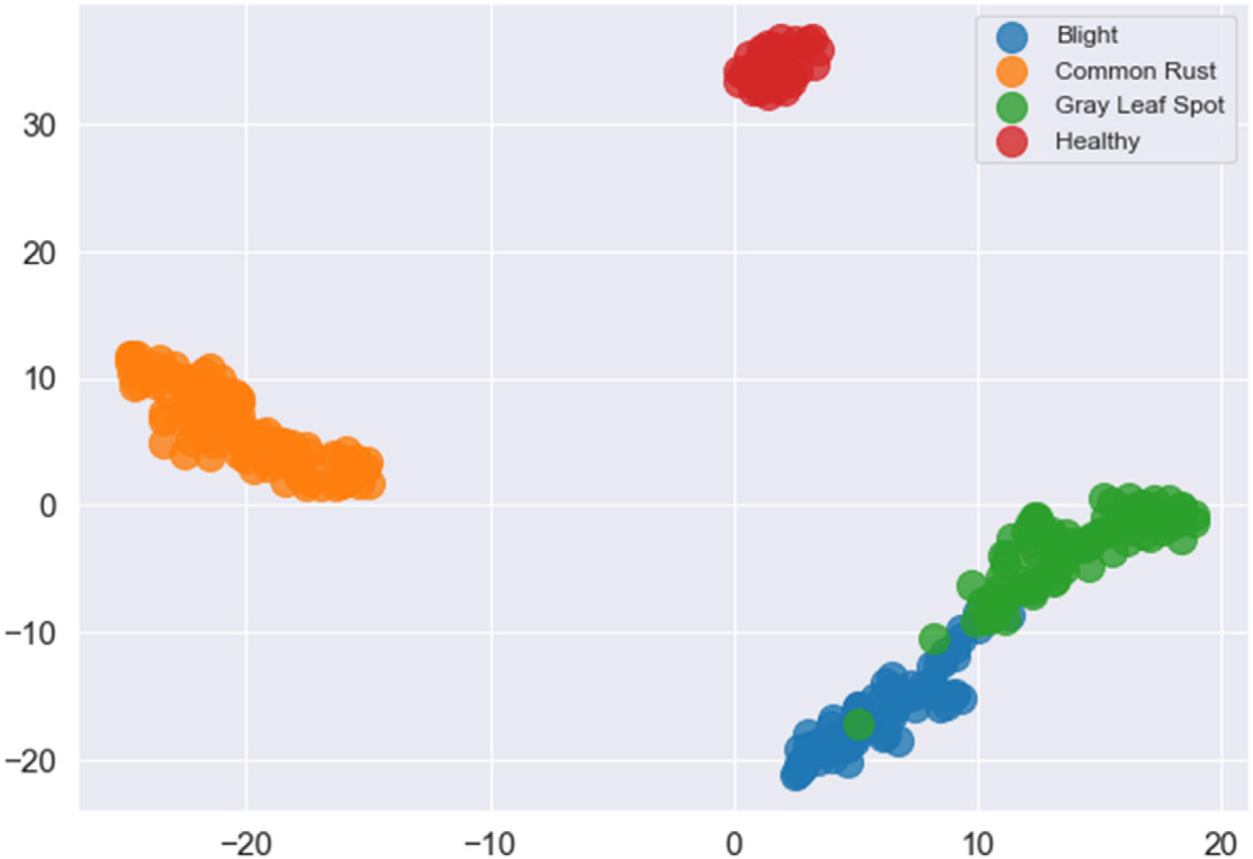
t-SNE graph of the model.

Figs. 6 and 7 illustrate the Training and Validation Loss and Training and Validation Accuracy. The graphs show a steady reduction in training and validation loss over the epochs, with training accuracy nearing 100 % and validation accuracy stabilizing around 98.25 % by the end of training. This indicates effective learning without overfitting. Fig. 8 presents the Receiver Operating Characteristic Curve (ROC), which exhibits how the model maintains its ability to balance true positives and false positives. The ROC curve shows an Area Under the Curve (AUC) of 0.98, demonstrating excellent classification performance across all classes. This reinforces the model's ability to distinguish between disease categories reliably. The PRC is also generated to further analyse the performance in class imbalances, with consistently high precision at various thresholds. The learned feature embeddings are visualized in Fig. 9 by creating t-SNE plots for the feature separability between disease classes. These plots show that classes are clustered. The model effectively focused on diseased regions by integrating attention mechanisms. This helped in distinguishing more accurately between visually similar symptoms. The ROC, PRC, and t-SNE analysis further validated the model's robustness, separability, and generalization capabilities. This highlights the significance of attention mechanisms with a pre-trained VGG16 model and prototypical few-shot learning in enhancing feature extraction for small, few-shot datasets.

The comparison presented in Table 2 highlights the superior performance of the proposed model over baseline CNN architectures such as ResNet-50, MobileNetV2, and DenseNet121. Although ResNet-50 reached an accuracy of 92.34 % with relatively low precision at 91.87 % and recall at 91.45 %, MobileNetV2 and DenseNet121 outperformed these, respectively at 94.12 % and 93.87 %. Still, the proposed method surpassed all the baseline models with an accuracy of 98.25 %, precision of 98.37 %, recall of 98.43 %, and F1-score of 98.39 %. Its high PRC-AUC (97.98 %) and ROC-AUC (99.12 %) scores indicate that it is strong in the effective management of class imbalances and in distinguishing true positives from false positives. The performance improvement is due to integrating few-shot learning with a meta-learning framework, attention mechanisms, and a pre-trained VGG16 backbone. These advancements enable the model to generalize to new disease instances and extract discriminative features from limited data. The results show the potential of the proposed model in revolutionizing corn disease detection, addressing the issues of data scarcity and class imbalance, and paving the way for efficient, scalable, and reliable solutions in precision agriculture.

**Table 2:** Comparison of the proposed model with the baseline models.

Model	Accuracy (%)	Precision (%)	Recall (%)	F1-Score (%)	PRC-AUC (%)	ROC-AUC (%)
ResNet-50	92.34	91.87	91.45	91.66	89.23	93.01
MobileNetV2	94.12	93.89	94.00	93.94	90.87	94.45
DenseNet121	93.87	93.45	93.67	93.56	90.22	94.20
**Proposed Method**	**98.25**	**98.37**	**98.43**	**98.39**	**97.98**	**99.12**

To ensure the robustness and generalization capabilities of the proposed model, extensive evaluations are performed across diverse datasets. This evaluation introduced an independent external dataset, i.e., a query set, other than the training and validation data for assessing generalizability to unseen distributions. High accuracy and consistency in performance without much degradation compared to primary dataset results further support the claim of this approach to handle a range of real-world scenarios. Synthetic samples are generated using Generative Adversarial Networks (GANs) to address diversity further. To this end, the GAN-based augmentation strategy is performed primarily to increase underrepresented categories and inject variability within the training data. This facilitated an improved model that could distinguish between visually similar disease categories and adept at handling class imbalances, as confirmed by higher recall and F1 scores across all classes. The cross-validation strategy is utilized for cross-evaluation across data splits. Such practice gives adequate exposure to average performance across numerous experiments with datasets for a reassessment of the reliability of the proposed model without probable overfitting risks. Therefore, the diversity of datasets may enrich learning features with more possible and actual performance adaptations that can be applied to agriculture practice scenarios. Results for testing the model against the external dataset, GAN-based augmentation, and cross-validation experiments are tabulated in detail in Table 3. It shows the generalization ability of the proposed model on new data distributions, captured along with higher diversity within datasets.

**Table 3:** Dataset diversity and generalization testing results.

Experiment	Accuracy (%)	Precision (%)	Recall (%)	F1-Score (%)	Comments
Primary Dataset (Validation)	98.25	98.37	98.43	98.39	Results on the primary dataset used for training and validation.
External Dataset	97.62	97.45	97.80	97.62	The model was tested on an unseen dataset to evaluate generalization.
GAN Augmented Data	98.75	98.61	98.89	98.75	Results with synthetic data added to enhance class diversity.
Cross-Validation (Average)	98.10	98.21	98.33	98.26	Average performance across 5-fold cross-validation.

The proposed method achieves state-of-the-art performance in diagnosing corn diseases, providing a scalable solution for agricultural applications where labeled data is limited. This work addresses the limitations of traditional classification methods by enabling effective disease identification with minimal labeled data. The insights gained from this research highlight the adaptability and efficiency of the proposed framework in learning from diverse disease instances and quickly generalizing to new disease types or environmental variations. Our findings demonstrate the model's potential to enhance precision agriculture practices by mitigating the impact of emerging disease threats and ensuring sustainable maize production. This work contributes to advancing the field of automated crop disease management by showcasing the practicality of few-shot learning techniques in agricultural applications. Future extensions of this study could involve integrating multi-modal data and real-time monitoring systems and applying this framework to other crops, further broadening its scope and impact.

## Limitations

Not applicable.

## Ethics statements

This research did not involve research on humans or animals, and no data is involved from social media platforms.

## CRediT authorship contribution statement

**Ruchi Rani:** Writing – original draft, Validation, Visualization, Methodology. **Jayakrushna Sahoo:** Supervision, Writing – review & editing. **Sivaiah Bellamkonda:** Supervision, Writing – review & editing. **Sumit Kumar:** Writing – review & editing, Funding acquisition.

## Declaration of competing interest

The authors declare that they have no known competing financial interests or personal relationships that could have appeared to influence the work reported in this paper.

## Data Availability

The corn/maize dataset used in this study is taken from Roboflow Universe and Kaggle 1. https://www.kaggle.com/datasets/smaranjitghose/corn-or-maize-leaf-disease-dataset 2. https://universe.roboflow.com/final-enlye/corn-disease.
